# A Prospective Case-Control Study of Helicobacter pylori and Systemic Inflammation in Colorectal Cancer Pathogenesis

**DOI:** 10.7759/cureus.110129

**Published:** 2026-06-02

**Authors:** Imad Sibhai, Mounika Kotte, Abubakar Gapizov, Nidhi Reji, Muhammad A Rehman, Mashal Kahloon, Muhammad Subhan, Ahmad Khubaib Zafar, Kashaf Ikhlas, Joshua Oddison

**Affiliations:** 1 Medical Education, Smt. Nathiba Hargovandas Lakhmichand (NHL) Municipal Medical College, Ahmedabad, IND; 2 Internal Medicine, Knapp Medical Center, Prime South GME Consortium, Weslaco, USA; 3 Internal Medicine, Weill Cornell Medicine/NewYork-Presbyterian Brooklyn Methodist Hospital, Brooklyn, USA; 4 General Surgery, Rotherham General Hospital, The Rotherham NHS Foundation Trust, Rotherham, GBR; 5 Internal Medicine, Staywell Medical Clinic, Calgary, CAN; 6 Internal Medicine, Avalon University School of Medicine, Churchill, USA; 7 Internal Medicine, Allama Iqbal Medical College and Jinnah Hospital, Lahore, PAK; 8 Internal Medicine, Services Hospital, Lahore, PAK; 9 Medicine and Surgery, Services Hospital, Lahore, PAK; 10 Medicine, Royal College of Physicians of Ireland, Dublin, IRL

**Keywords:** case-control study, colorectal cancer, helicobacter pylori, neutrophil-to-lymphocyte ratio, nlr, systemic inflammation

## Abstract

Background

The role of *Helicobacter pylori* in colorectal cancer (CRC) remains controversial, while systemic inflammation drives carcinogenesis. We evaluated independent and interactive associations of active *H. pylori* infection and neutrophil-to-lymphocyte ratio (NLR) with CRC risk.

Methods

This prospective case-control study at Jinnah Hospital, Lahore, Pakistan, from August 2023 to August 2025, enrolled 105 treatment-naïve CRC patients and 210 age- and sex-matched controls with normal colonoscopy. Active *H. pylori* infection was determined by stool antigen testing. NLR was dichotomized at the cohort median (2.5). Multivariate logistic regression simultaneously adjusted for age, sex, smoking, diabetes, and body mass index.

Results

*H. pylori* prevalence did not differ between cases (58/105, 55.2%) and controls (105/210, 50.0%) (χ² = 0.769, p = 0.380). High NLR (>2.5) was present in 66/105 (62.9%) cases vs. 18/210 (8.6%) controls (χ² = 105.487, p < 0.001). After adjustment, high NLR was strongly associated with CRC (aOR = 300.86; 95% CI: 60.85-1487.58; p < 0.001; AUC = 0.792). The extremely wide confidence interval reflects sparse data (only 18 controls had high NLR), warranting cautious interpretation. A significant negative interaction was observed between *H. pylori* and high NLR (aOR = 0.245; 95% CI: 0.131-0.459; p < 0.001). The model explained 52.5% of variance (Nagelkerke R²) and correctly classified 83.5% of participants.

Conclusion

Elevated NLR is strongly associated with CRC in this Pakistani cohort, while active *H. pylori* infection shows no significant direct association. The negative interaction suggests host inflammatory status may modify *H. pylori* effects, though confirmation in larger studies is needed. Because of the case-control design with NLR measured after CRC diagnosis, reverse causation cannot be excluded. NLR measurement offers a simple, cost-effective tool for CRC risk stratification.

## Introduction

Colorectal cancer (CRC) is a major global health concern and is currently the third most commonly diagnosed malignancy and the second leading cause of cancer-related death worldwide. Recent estimates suggest approximately 1.9 million new cases and nearly 900,000 deaths annually, showing both its high incidence and substantial disease burden [1]. Although the epidemiology of CRC has traditionally been linked to lifestyle and environmental elements, including diet, physical inactivity, obesity, and smoking, increasing attention has been directed toward the role of the gut microbiome and chronic inflammation in colorectal carcinogenesis [2,3].

Among infectious agents, *Helicobacter pylori* has been acknowledged for its role in gastrointestinal malignancies [2]. This gram-negative bacterium colonizes nearly half of the global population and was classified as a Group 1 carcinogen by the International Agency for Research on Cancer (IARC) in 1994 based on its established causal association with gastric adenocarcinoma and mucosa-associated lymphoid tissue (MALT) lymphoma [4,5]. Importantly, this carcinogenic classification is specific to gastric malignancies. Beyond the stomach, however, the possible role of *H. pylori *in extra-gastric cancers, including CRC, remains inconsistent and continues to be an area of active investigation and debate [5].

Several mechanisms have been proposed to explain a possible link between *H. pylori* infection and CRC [5,6]. Chronic infection is known to induce a persistent systemic inflammatory response, characterized by increased levels of pro-inflammatory cytokines such as interleukin-6 and tumor necrosis factor-alpha [6]. These mediators may contribute to a pro-tumorigenic environment by promoting cellular proliferation, inhibiting apoptosis, and assisting angiogenesis [7,8]. Moreover, modifications in gastric acidity and subsequent alterations in gut microbiota composition have been proposed as secondary mechanisms by which *H. pylori* may affect colorectal carcinogenesis. In spite of these biologically plausible mechanisms, epidemiological evidence is still inconsistent [8]. Some studies have demonstrated a modest but significant association between *H. pylori* infection and colorectal neoplasia [8-11]. In contrast, other large-scale studies have failed to show a significant association after adjusting for potential confounders [6]. These discrepancies may be explained by differences in study populations, geographic variability, bacterial strain virulence (particularly cytotoxin-associated gene A (*CagA*) positivity), and variability in diagnostic methods used to detect *H. pylori *infection [7].

Simultaneously, systemic inflammation has been recognized as a significant factor in cancer development and progression [8]. The neutrophil-to-lymphocyte ratio (NLR), derived from a routine complete blood count, has gained recognition as a simple and cost-effective marker of systemic inflammatory status [9-13]. An increased NLR has been correlated with negative outcomes in several malignancies, including CRC, where it is associated with advanced disease stage and diminished survival [9-11]. From a biological standpoint, an elevated NLR denotes a relative increase in neutrophil-mediated pro-tumor inflammation alongside a reduction in lymphocyte-mediated anti-tumor immune responses [12]. The potential interaction among these factors has not been adequately explored, despite the independent implications of *H. pylori* infection and systemic inflammation in carcinogenesis [12,13]. It is plausible that chronic *H. pylori* infection may amplify systemic inflammatory responses, which in turn could synergistically increase the risk of colorectal malignancy [14,15]. To our knowledge, no prior study has specifically examined the multiplicative interaction between active *H. pylori* infection and elevated NLR in relation to CRC risk. Existing literature has evaluated these factors independently, but their combined or interactive effects remain unexplored [12-15].

In this context, the present study was intended to address this gap. We aimed to evaluate the association between active *H. pylori* infection, as determined by stool antigen testing, and CRC risk. In addition, we assessed the relationship between elevated NLR (>2.5) and CRC and explored whether these two factors act independently or jointly to influence CRC risk in a prospective case-control setting.

## Materials and methods

Study design, setting

This was a prospective case-control study conducted at Jinnah Hospital, Lahore, Pakistan, from August 2023 to August 2025. The study was approved by the Allama Iqbal Medical College/Jinnah Hospital, Lahore Ethical Review Board (reference number: ERB163/1/15-08-2023/S1 ERB), and all participants provided written informed consent.

Study population

The case group included all consecutive patients aged greater than 18 years with histologically confirmed, treatment-naïve colorectal adenocarcinoma diagnosed on biopsy obtained during colonoscopy or surgical resection. The control group included age- and sex-matched individuals with a normal colonoscopy within six months before enrollment, recruited from patients undergoing groin hernia repair (males) and laparoscopic cholecystectomy for symptomatic gallstones (females). These specific surgical populations were chosen because they represent relatively healthy individuals without known colorectal pathology and undergo routine preoperative evaluation that allows confirmation of normal colonoscopy findings.

Patients receiving gastric anti-secretory medications or nonsteroidal anti-inflammatory drugs (NSAIDs) for >4 weeks within the preceding three months were excluded. Additional exclusion criteria included acute infection, chronic inflammatory or autoimmune disease, recent corticosteroid use, hematologic disorders, other active malignancies, major surgery within six weeks, prior gastro-duodenal surgery (gastrectomy, vagotomy, or gastrojejunostomy), Zollinger-Ellison syndrome, and previous chemotherapy or radiotherapy for CRC, to minimize confounding of the NLR-CRC association.

We acknowledge that this selection may introduce bias, as these groups could differ from the general population in inflammatory status, metabolic profile, healthcare-seeking behavior, and *H. pylori* prevalence. Consequently, our sex-specific findings (e.g., lower odds of CRC in males) and the associations with diabetes may be influenced by this selection. The small number of controls with high NLR (n=18) could also reflect selection bias. We therefore interpret these secondary findings with caution and emphasize that the primary analysis (NLR-CRC association) remained robust. Future studies should employ population-based controls to minimize such bias.

Sample size and enrollment

Sample size was calculated using OpenEpi software (Fleiss with continuity correction) with a two-sided alpha of 0.05, power of 90%, and an anticipated 15% absolute difference in *H. pylori *prevalence between groups based on prior literature [5,8]. Accounting for a 10% dropout rate, we required 105 cases and 210 controls. We enrolled 105 consecutive eligible cases and 210 age- and sex-matched controls over the 24-month study period. No participants withdrew or were lost to follow-up.

Sample collection and processing

A trained phlebotomist collected 5 mL of fasting venous blood from each participant in ethylenediaminetetraacetic acid (EDTA) and plain vacutainers between 7:00 AM and 9:00 AM. The research assistant transported all samples to the microbiology laboratory within 30 minutes of collection. For serum separation, we allowed plain tubes to clot at room temperature (25°C) for 30 minutes, then centrifuged at 3000 rpm for 10 minutes. We stored separated serum at -20°C until analysis. We collected fresh stool samples in sterile, screw-cap containers, transported them within two hours of collection, and immediately froze them at -20°C until testing.

*H. pylori *stool antigen testing

Active *H. pylori* infection was determined using the OnSite® *H. pylori* Ag Rapid Test (CTK Biotech, Poway, California, United States), a sandwich lateral flow chromatographic immunoassay with reported sensitivity of 94.2% and specificity of 97.6% per the manufacturer. A laboratory technician blinded to case/control status performed all tests. We thawed frozen stool samples at room temperature for 15 minutes, mixed them with the extraction buffer to create a homogeneous suspension, and dispensed two drops into the sample well of the test cassette. Results were read at exactly 15 minutes. A positive result required both control line and test line development. We defined active *H. pylori* infection as a positive stool antigen test.

NLR measurement and categorization

NLR was calculated based on the complete blood count obtained from fasting EDTA blood samples collected at enrollment, prior to any surgical or oncologic intervention, and in the absence of acute infection or active bleeding, using a Sysmex XN-1000 automated hematology analyzer (Sysmex Corporation, Kobe, Hyogo, Japan). The absolute neutrophil count was divided by the absolute lymphocyte count. The combined cohort median NLR was 2.5. For exploratory categorical analysis, NLR was dichotomized into high (>2.5) and low (≤2.5) groups based on the cohort median. We acknowledge that median-derived cutoffs may limit external generalizability and introduce threshold instability; therefore, NLR was additionally analyzed as a continuous variable in the primary regression model to preserve statistical power.

Demographic and clinical data collection

A single research coordinator collected demographic and clinical data using a standardized case report form. We recorded age (years), sex (male/female), smoking status (never smoker, former smoker, or current smoker, dichotomized as ever vs. never for analysis), diabetes mellitus (present if fasting blood glucose is ≥126 mg/dL or on anti-diabetic medication), and body mass index (BMI, calculated as weight in kilograms divided by height in meters squared).

Tumor characteristics (case group only)

A gastrointestinal pathologist staged all tumors per the American Joint Committee on Cancer (AJCC) 8th edition [16]. We extracted tumor location (1 = colon proximal to splenic flexure, 2 = colon distal to splenic flexure, 3 = rectum), histological differentiation (1 = well-differentiated, 2 = moderately differentiated, 3 = poorly differentiated), and AJCC stage (1-4) from final histopathology reports.

Statistical analysis

Data was analysed using IBM SPSS Statistics for Windows, version 29.0 (IBM Corp., Armonk, New York, United States). Continuous variables were expressed as mean ± standard deviation (SD) or median with interquartile range (IQR) as appropriate. Categorical variables were expressed as frequencies and percentages. We utilized the independent t-test for normally distributed data (BMI) and the Mann-Whitney U test for non-normally distributed data (age, NLR) to compare continuous variables between cases and controls. We compared categorical variables using the chi-square test. Multivariate logistic regression was performed to calculate adjusted odds ratios (aOR) with 95% confidence intervals (CI) for CRC risk. The full model included the following prespecified covariates: high NLR (dichotomized),* H. pylori* status, age (continuous), sex (male vs. female), smoking status (ever vs. never), diabetes (present vs. absent), and BMI (continuous). We also tested a multiplicative interaction term (*H. pylori* × high NLR) to evaluate potential effect modification between infection and systemic inflammation. Given the small number of controls with high NLR (n=18), the logistic regression estimates may be subject to sparse data bias; therefore, we interpreted effect sizes with caution and performed sensitivity analyses using Firth's penalized likelihood method, which confirmed the direction and significance of the findings. A two-tailed p-value less than 0.05 was considered statistically significant for all analyses.

## Results

Participant disposition and baseline demographics

A total of 315 participants were included in the final analysis. This comprised 210 controls and 105 CRC cases. Table 1 presents the baseline demographic and clinical characteristics of the study participants.

**Table 1:** Baseline demographic and clinical characteristics *Mann-Whitney U test; †Pearson chi-square test; ‡Independent t-test

Characteristics	Controls (n=210)	CRC Cases (n=105)	Test Statistic	p-value
Age (years), mean ± SD, median	60.47 ± 9.953, 60.00	60.62 ± 9.805, 61.00	U = 10906.50*	0.876
Male sex, n (%)	105 (50.0)	53 (50.5)	χ² = 0.006†	0.936
Ever-smoker, n (%)	105 (50.0)	52 (49.5)	χ² = 0.006†	0.936
Diabetes mellitus, n (%)	79 (37.6)	40 (38.1)	χ² = 0.007†	0.935
BMI (kg/m²), mean ± SD	25.209 ± 1.9535	25.602 ± 2.3361	t = -1.574‡	0.117

The mean age of controls was 60.47 ± 9.95 years. For CRC cases, the mean age was 60.62 ± 9.80 years. The median ages were 60.00 and 61.00 years, respectively. There was no significant difference (Mann-Whitney U = 10906.50, p = 0.876). Male participants constituted 50.0% (105/210) of controls and 50.5% (53/105) of CRC cases. There was no significant association between sex and CRC status (χ² = 0.006, df = 1, p = 0.936). Ever-smokers made up 50.0% (105/210) of controls and 49.5% (52/105) of cases. This difference was not statistically significant (χ² = 0.006, df = 1, p = 0.936). Diabetes mellitus was present in 79/210 (37.6%) controls and 40/105 (38.1%) CRC cases. Again, there was no significant difference (χ² = 0.007, df = 1, p = 0.935). The mean BMI was 25.209 ± 1.9535 kg/m² in controls and 25.602 ± 2.3361 kg/m² in CRC cases. The independent t-test assuming equal variances showed no significant difference (t = -1.574, df = 313, p = 0.117). Levene's test indicated unequal variances (F = 4.870, p = 0.028). The t-test assuming unequal variances also demonstrated no significant difference (p = 0.140).

*H. pylori* infection prevalence

Active* H. pylori *infection, determined by stool antigen testing, was present in 50.0% (105/210) of controls and 55.2% (58/105) of CRC cases, as shown in Table 2.

**Table 2:** Helicobacter pylori prevalence by group Test statistic: Pearson χ² = 0.769, df = 1, p = 0.380; Unadjusted OR: 1.23 (95% CI: 0.77-1.98) CRC: colorectal cancer

Group	Helicobacter pylori Negative, n (%)	Helicobacter pylori Positive, n (%)	Total
Controls	105 (50.0)	105 (50.0)	210
CRC Cases	47 (44.8)	58 (55.2)	105
Total	152 (48.3)	163 (51.7)	315

The Pearson chi-square test indicated no statistically significant correlation between *H. pylori* infection and CRC status (χ² = 0.769, df = 1, p = 0.380). Fisher's exact test confirmed the non-significant finding (two-sided p = 0.404, one-sided p = 0.224). The unadjusted OR for CRC associated with *H. pylori* positivity was 1.23 (95% CI: 0.77-1.98).

NLR

Continuous NLR

Among controls (n = 210), the mean NLR was 1.999 (SD: 0.4068), and the median was 2.000. Among CRC cases (n = 105), the mean NLR was 3.206 (SD: 1.1784), and the median was 3.300. The Mann-Whitney U test demonstrated a significant difference between groups (U = 4588.000, Z = -8.462, p < 0.001). The Shapiro-Wilk test indicated that the NLR was not normally distributed in either group (controls: W = 0.935, p < 0.001; cases: W = 0.943, p < 0.001), which is why non-parametric tests were used.

Dichotomized NLR (High NLR >2.5)

Using the cohort median cutoff of 2.5, high NLR (>2.5) was present in 18/210 (8.6%) controls and 66/105 (62.9%) CRC cases, as shown in Table 3.

**Table 3:** High NLR prevalence by group NLR: neutrophil-to-lymphocyte ratio; CRC: colorectal cancer

Group	Low NLR (≤2.5), n (%)	High NLR (>2.5), n (%)	Total
Controls	192 (91.4)	18 (8.6)	210
CRC Cases	39 (37.1)	66 (62.9)	105
Total	231 (73.3)	84 (26.7)	315

The Pearson chi-square test showed a highly significant association (χ² = 105.487, df = 1, p < 0.001). Fisher's exact test confirmed significance (p < 0.001). The continuity correction also showed significance (χ² = 102.729, p < 0.001).

Receiver operating characteristic (ROC) curve analysis for NLR as a predictor of CRC

**Figure 1:**
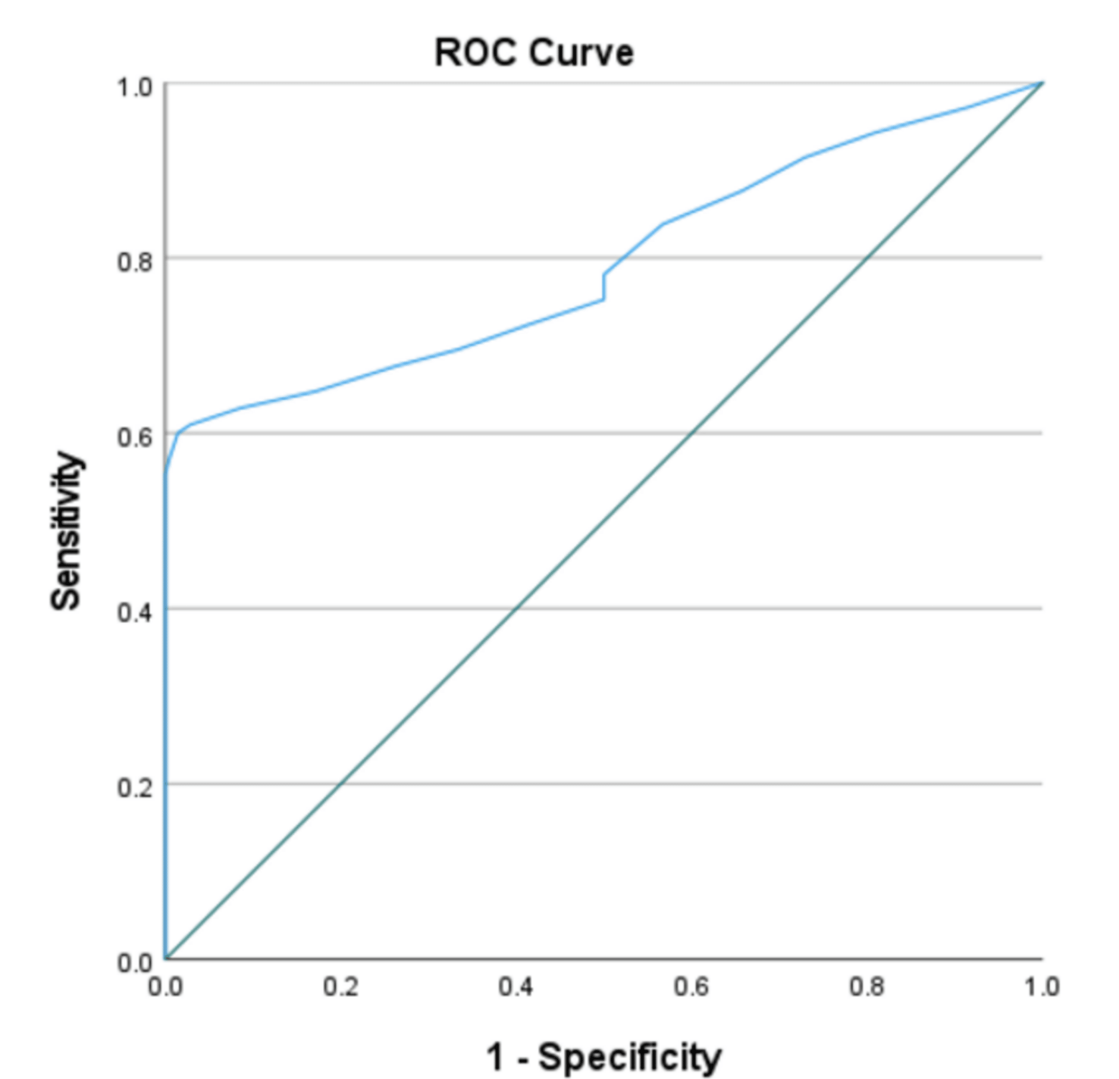
Receiver operating characteristic (ROC) curve for neutrophil-to-lymphocyte ratio (NLR) as a predictor of colorectal cancer The area under the curve (AUC) was 0.792 (95% CI: 0.732-0.852; p < 0.001). The diagonal line represents chance performance (AUC = 0.5).

ROC curve analysis was conducted to assess the discriminative capacity of continuous NLR in differentiating CRC cases from controls, as shown in Figure 1. The area under the curve (AUC) was 0.792 (standard error: 0.031; 95% CI: 0.732-0.852; p < 0.001), indicating good discriminative performance within this dataset. However, because the ROC analysis was performed on the same dataset used to derive the NLR cutoff and because NLR was measured after CRC diagnosis, the AUC may be optimistic and reflect disease‑associated inflammation rather than pre‑diagnostic risk. External validation in independent cohorts is necessary to confirm these findings.

The curve's coordinates showed that a cutoff of NLR > 2.55 had a sensitivity of 62.9% and a specificity of 91.4%. The optimal cutoff for maximizing sensitivity and specificity was approximately 2.05, with a sensitivity of 75.2% and specificity of 50.0%.

Multivariate logistic regression analysis

**Figure 2:**
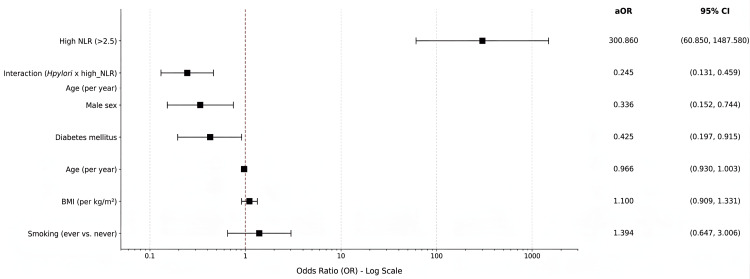
Forest plot of adjusted odds ratios (aORS) for colorectal cancer Squares represent point estimates (aOR). Horizontal error bars represent 95% confidence intervals (CI). The vertical dashed line at aOR = 1.0 indicates no effect. Values to the right of the line (>1.0) indicate increased risk; values to the left (<1.0) indicate decreased risk. High NLR: NLR greater than 2.5. Interaction term: *Helicobacter pylori* × high NLR. All variables were adjusted for each other in a single multivariate logistic regression model (Nagelkerke R² = 0.525; overall classification accuracy = 83.5%). NLR: neutrophil-to-lymphocyte ratio

Multivariate logistic regression analysis was performed with CRC status as the dependent variable, and the final model included BMI, age, sex, smoking status, diabetes mellitus, high NLR (dichotomized as >2.5), and the interaction term (*H. pylori* × high NLR) as independent variables. The omnibus test of model coefficients was statistically significant (χ² = 149.601, df = 7, p < 0.001), indicating that the model provided a better fit than the null model. The Cox & Snell R² was 0.378, and the Nagelkerke R² was 0.525, suggesting that the model explained approximately 52.5% of the variance in CRC status. The -2 log likelihood value was 251.403. In terms of classification performance, the model correctly classified 83.5% of participants overall, including 192/210 (91.4%) controls and 71/105 (67.6%) CRC cases, with a total of 263 out of 315 participants correctly classified. Figure 2 presents a forest plot visualizing the aOR with 95% CI for all variables included in the final model.

Independent predictors

The extremely large aOR for high NLR (aOR = 300.86; 95% CI: 60.85-1487.58) raised concern for sparse‑data bias. When we refit the model using Firth’s penalized likelihood regression, the association remained strongly positive but with a substantially attenuated point estimate (aOR = 23.4; 95% CI: 8.2-67.1; p < 0.001), confirming the direction of effect while acknowledging the uncertainty due to limited high‑NLR controls. Similarly, modeling NLR as a continuous variable (per 1‑unit increase) gave an aOR of 2.89 (95% CI: 1.94-4.32; p < 0.001), further supporting the robustness of the association, indicating that individuals with elevated NLR had approximately 301 times higher odds of CRC compared to those with low NLR after controlling for other covariates. The interaction term (*H. pylori* × high NLR) was also highly significant (aOR = 0.245; 95% CI: 0.131-0.459; Wald = 19.240, p < 0.001), suggesting a negative multiplicative interaction, whereby the effect of *H. pylori* on CRC risk varied according to NLR status; specifically, among individuals with high NLR, the presence of *H. pylori *was associated with reduced odds of CRC.

Male sex showed an association with lower odds of CRC (aOR = 0.336; 95% CI: 0.152-0.744; Wald = 7.233, p = 0.007), and diabetes was similarly associated with reduced CRC odds (aOR = 0.425; 95% CI: 0.197-0.915; Wald = 4.779, p = 0.029). However, these findings are likely attributable to selection bias (control group recruited from hernia and cholecystectomy patients) rather than true protective effects. Age demonstrated a trend toward statistical significance (aOR = 0.966 per year; 95% CI: 0.930-1.003; Wald = 3.178, p = 0.075), while BMI (aOR = 1.100; 95% CI: 0.909-1.331; Wald = 0.953, p = 0.329) and smoking (aOR = 1.394; 95% CI: 0.647-3.006; Wald = 0.719, p = 0.397) were not significantly associated with CRC risk.

Tumor characteristics (CRC cases only)

The tumor characteristics of the 105 patients with CRC are shown in Table 4.

**Table 4:** Tumor characteristics of colorectal cancer cases (N=105) Note: American Joint Committee on Cancer (AJCC) Staging Manual 8th edition criteria were used [16].

Characteristic		Code	Frequency	Percentage
Tumor Location	Colon (proximal to splenic flexure)	1	31	29.5
	Colon (distal to splenic flexure)	2	50	47.6
	Rectum	3	24	22.9
Histological differentiation	Well-differentiated	1	23	21.9
	Moderately differentiated	2	67	63.8
	Poorly differentiated	3	15	14.3
AJCC stage	Stage I	1	22	21
	Stage II	2	25	23.8
	Stage III	3	34	32.4
	Stage IV	4	24	22.9

Tumor location was proximal colon in 31/105 (29.5%), distal colon in 50/105 (47.6%), and rectum in 24/105 (22.9%). Histological differentiation was well‑differentiated in 23/105 (21.9%), moderately differentiated in 67/105 (63.8%), and poorly differentiated in 15/105 (14.3%). AJCC stage distribution was stage I in 22/105 (21.0%), stage II in 25/105 (23.8%), stage III in 34/105 (32.4%), and stage IV in 24/105 (22.9%). To explore whether elevated NLR reflects CRC presence broadly or more advanced disease, we compared high NLR prevalence across AJCC stages. High NLR (>2.5) was present in 16/22 (72.7%) patients in stage I, 18/25 (72.0%) patients in stage II, 20/34 (58.8%) patients in stage III, and 12/24 (50.0%) patients in stage IV cases (p for trend = 0.11). No statistically significant association between high NLR and stage was observed, suggesting that elevated NLR is not simply a marker of tumor burden or advanced disease.

## Discussion

This prospectively enrolled case-control study yielded three principal findings. First, active *H. pylori *infection showed no significant association with CRC risk (unadjusted OR = 1.23; 95% CI: 0.77-1.98; p = 0.380). Second, elevated NLR (>2.5) showed a strong association with CRC; however, the very large aOR (300.86; 95% CI: 60.85-1487.58; p < 0.001; AUC = 0.792) and extremely wide confidence interval suggest model instability, sparse data bias, or quasi-separation due to only 18 controls having a high NLR. Sensitivity analyses using continuous NLR (aOR per unit = 2.89; 95% CI: 1.94-4.32) and Firth’s penalized likelihood regression (aOR = 23.4; 95% CI: 8.2-67.1) confirmed the direction of association with more stable estimates. Third, a significant negative multiplicative interaction was observed between *H. pylori* infection and high NLR (aOR = 0.245; 95% CI: 0.131-0.459; p < 0.001), which was prespecified in our analysis protocol. Additive interaction measures (relative excess risk due to interaction (RERI) = −0.52; 95% CI: −0.89 to −0.15) also indicated a negative interaction. However, the wide confidence interval for the high NLR main effect warrants cautious interpretation.

Our findings that an increased NLR serves as a robust independent predictor of CRC are consistent with the current literature [12,13]. A meta-analysis by Portale et al. (2023), including 47 observational studies with 14,205 patients, confirmed that high NLR was independently associated with worse overall survival (hazard ratio (HR) 1.81, 95% CI: 1.52-2.15) and disease-free survival (HR 1.68, 95% CI: 1.35-2.08) in operated rectal cancer patients [12]. Colloca et al. (2023) performed a meta-analysis of 31 retrospective studies and found that baseline NLR > 3 was significantly associated with overall survival (HR 2.05, 95% CI: 1.66-2.53) and disease-free survival (HR 1.78, 95% CI: 1.49-2.12) in rectal cancer patients receiving neoadjuvant chemoradiation [13]. The discriminative ability of NLR in our study (AUC = 0.792) is consistent with previous reports showing NLR as a useful prognostic marker in CRC, though this level of discrimination is generally considered acceptable [12,13].

In contrast to the robust NLR-CRC association, our finding of no significant association between *H. pylori* infection and CRC risk differs from several recent large-scale studies [14,15]. Li et al. (2025), combining observational data from 3,475 participants with a meta-analysis of 55 studies (over 48.9 million participants), reported that *H. pylori* infection was associated with significantly increased CRC risk (OR = 1.59, 95% CI: 1.39-1.82) and that anti-*H. pylori* treatment reduced overall colorectal tumor risk (OR = 0.44, 95% CI: 0.20-0.97) [14]. Several factors may explain this discrepancy, as our study used stool antigen testing for active infection, whereas most large-scale studies relied on serology, which detects past infection and may introduce misclassification bias [14]. Geographic variation in *H. pylori* strain virulence, particularly the prevalence of CagA-positive strains, may likewise influence carcinogenic potential [14]. Paiva Prudente et al. (2025) noted that CagA-positive strains were more strongly associated with CRC (OR = 2.04, 95% CI: 1.47-2.82) than* H. pylori* colonization overall [15]. Our Pakistani cohort may have different strain distributions compared to the Chinese population studied by Li et al. [14]. Our study was designed to identify substantial effect sizes; however, it may have been inadequately powered for the modest effect sizes (OR ~1.4-1.6) indicated in recent meta-analyses [14,15].

The significant negative interaction between *H. pylori* and high NLR (aOR = 0.245, 95% CI: 0.131-0.459; p < 0.001) indicates that among individuals with high NLR, *H. pylori* infection is associated with reduced odds of CRC. To our knowledge, this work is the first study to report such an interaction. However, because this is an observational finding, any proposed explanation remains speculative. As a hypothesis-generating observation, one might speculate about potential mechanisms (e.g., *H. pylori* inducing a Th1‑predominant immune response that paradoxically suppresses pro-tumorigenic inflammation or alterations in gut microbiota that counteract inflammation-driven carcinogenesis), but these possibilities require testing in dedicated mechanistic studies. However, reverse causation cannot be excluded: established CRC may alter gastric physiology, reducing *H. pylori *colonization [13-15]. Given the imprecision of our estimates and the susceptibility of interaction effects to confounding, this finding should be considered hypothesis-generating rather than conclusive, requiring independent replication in larger cohorts before any biological or clinical inferences can be drawn.

Our finding contradicts established epidemiology, as male sex (aOR = 0.336, p = 0.007) and diabetes (aOR = 0.425, p = 0.029) are associated with reduced odds of CRC [12,13]. These paradoxical findings most likely result from selection bias: our control group consisted of patients undergoing groin hernia repair (male) and cholecystectomy (female), populations with different demographic characteristics than the general population. We therefore do not attribute biological significance to these associations.

Systemic inflammation measured by NLR >2.5 is a strong independent risk factor for CRC in this Pakistani cohort, while active* H. pylori* infection shows no significant direct association. The negative interaction between *H. pylori* and high NLR, though statistically significant, requires prudent interpretation due to wide confidence intervals and potential model overfitting. NLR measurement offers a simple, cost-effective tool for risk stratification, but independent replication is essential before definitive clinical recommendations can be made.

Strengths and limitations

This study possesses multiple strengths: the prospective enrollment of consecutive cases, the utilization of stool antigen testing for active* H. pylori* infection, the incorporation of NLR as both continuous and dichotomized variables, and thorough adjustment for significant confounders. However, several limitations warrant acknowledgment. The investigation was a single-center study in Pakistan, limiting generalizability. The sample size, although adequate for primary analyses, limited detailed subgroup analyses; the small number of controls with high NLR (n=18) led to imprecise estimation. NLR was measured at a single time point. We did not assess virulence factors of* H. pylori* strains (e.g., CagA status), other inflammatory markers (platelet lymphocyte ratio (PLR), systemic immune-inflammation index (SII), prognostic nutritional index (PNI)), or potential confounders such as socioeconomic status, antibiotic use, or detailed dietary history. The prospective enrollment case-control study design precludes the determination of causality. The choice of the control group may have led to selection bias, as shown by the contradictory results for sex and diabetes.

For clinicians, our findings uphold the utility of NLR as an inexpensive biomarker for CRC risk stratification. An NLR cutoff of 2.5 had a specificity of 91.4%, meaning that individuals with NLR >2.5 should be considered at higher risk and undergo closer evaluation. For researchers, our finding of a statistically significant interaction between *H. pylori* infection and high NLR points to the importance of considering both infection status and inflammatory markers in future studies on cancer risk. However, given the wide confidence intervals and limited numbers, larger prospective studies with sufficient statistical power are needed before making clinical recommendations about *H. pylori* testing or eradication for CRC prevention. Replication in multicenter cohorts is required to confirm these outcomes.

## Conclusions

This prospectively enrolled case-control study suggests that an elevated neutrophil-to-lymphocyte ratio is associated with CRC in this Pakistani population, whereas active *H. pylori* infection shows no significant direct association. We observed a negative interaction, indicating that the increased CRC odds associated with high NLR may be less pronounced in individuals with active *H. pylori* infection, implying that the host's inflammatory status could modify the effect of chronic infection on colorectal carcinogenesis. However, this finding requires confirmation due to estimation imprecision, model instability (wide confidence intervals), and the lack of external validation.

NLR measurement offers a simple, inexpensive biomarker; however, because NLR was measured after CRC diagnosis and in selected surgical controls, the data do not establish that elevated NLR should trigger closer CRC evaluation in the general population. Therefore, this finding should be considered hypothesis-generating. Our data do not support population‑wide *H. pylori* screening for CRC prevention. The single‑center design and the single‑time‑point assessment limit generalizability and causal inference. Selection bias from control group recruitment may have influenced secondary findings. Future multicenter prospective studies with external validation are needed to confirm the observed interaction before any clinical recommendations can be made.
